# Molecular Diversity of *Trypanosoma cruzi* Detected in the Vector *Triatoma protracta* from California, USA

**DOI:** 10.1371/journal.pntd.0004291

**Published:** 2016-01-21

**Authors:** Lisa A. Shender, Michael D. Lewis, Daniel Rejmanek, Jonna A. K. Mazet

**Affiliations:** 1 Wildlife Health Center; One Health Institute; School of Veterinary Medicine, University of California, Davis, Davis, California, United States of America; 2 Department of Pathogen Molecular Biology, Faculty of Infectious and Tropical Diseases, London School of Hygiene and Tropical Medicine, Keppel Street, London, United Kingdom; Liverpool School of Tropical Medicine, UNITED KINGDOM

## Abstract

**Background:**

*Trypanosoma cruzi*, causative agent of Chagas disease in humans and dogs, is a vector-borne zoonotic protozoan parasite that can cause fatal cardiac disease. While recognized as the most economically important parasitic infection in Latin America, the incidence of Chagas disease in the United States of America (US) may be underreported and even increasing. The extensive genetic diversity of *T*. *cruzi* in Latin America is well-documented and likely influences disease progression, severity and treatment efficacy; however, little is known regarding *T*. *cruzi* strains endemic to the US. It is therefore important to expand our knowledge on US *T*. *cruzi* strains, to improve upon the recognition of and response to locally acquired infections.

**Methodology/Principle Findings:**

We conducted a study of *T*. *cruzi* molecular diversity in California, augmenting sparse genetic data from southern California and for the first time investigating genetic sequences from northern California. The vector *Triatoma protracta* was collected from southern (Escondido and Los Angeles) and northern (Vallecito) California regions. Samples were initially screened via sensitive nuclear repetitive DNA and kinetoplast minicircle DNA PCR assays, yielding an overall prevalence of approximately 28% and 55% for southern and northern California regions, respectively. Positive samples were further processed to identify discrete typing units (DTUs), revealing both TcI and TcIV lineages in southern California, but only TcI in northern California. Phylogenetic analyses (targeting *COII-ND1*, *TR* and *RB19* genes) were performed on a subset of positive samples to compare Californian *T*. *cruzi* samples to strains from other US regions and Latin America. Results indicated that within the TcI DTU, California sequences were similar to those from the southeastern US, as well as to several isolates from Latin America responsible for causing Chagas disease in humans.

**Conclusions/Significance:**

*Triatoma protracta* populations in California are frequently infected with *T*. *cruzi*. Our data extend the northern limits of the range of TcI and identify a novel genetic exchange event between TcI and TcIV. High similarity between sequences from California and specific Latin American strains indicates US strains may be equally capable of causing human disease. Additional genetic characterization of Californian and other US *T*. *cruzi* strains is recommended.

## Author Summary

*Trypanosoma cruzi* is a protozoan parasite that causes Chagas disease in humans and dogs and may eventually lead to mortalities related to cardiac failure. This parasite is most frequently transmitted by triatomine bug vectors, commonly called “kissing bugs.” Although Chagas disease is predominately acquired in Latin American countries, *T*. *cruzi* exists in wildlife and vectors in some parts of the United States of America (US), including regions of California. Within the US, occasional cases of locally acquired Chagas disease have been reported, and recent serological surveys indicate that *T*. *cruzi* exposure may be occurring more commonly than previously realized. However, relatively little molecular research has been performed on the *T*. *cruzi* strains present in the US, especially within California. In this study, we collected nearly 100 kissing bugs from regions of northern and southern California to determine the *T*. *cruzi* prevalence and genetic diversity for each region’s kissing bug population. We compared DNA sequences obtained in this study to those of several *T*. *cruzi* strains found in Latin America and the southeastern US. Based on our data, we conclude that Californian *T*. *cruzi* samples are closely related to strains found in Latin America known to be associated with human infections.

## Introduction

*Trypanosoma cruzi* is a protozoan parasite that, in both humans and dogs, may cause an insidious onset of fatal cardiac disease[1]. Known as Chagas disease, *T*. *cruzi* is the most economically important parasitic infection in Latin America, where an estimated 8–9 million people are living with the chronic disease [2]. The parasite is most commonly transmitted vectorially, by numerous species of triatomine bugs (commonly called “kissing bugs”), distributed from Chile and Argentina in South America to approximately 42.5 degrees northern latitude of the United States of America [3, 4]. Only seven authochthonous clinical cases of Chagas disease in humans have been officially documented in the United States despite the fact that nine endemic *Triatoma* species are known to harbor *T*. *cruzi* [1]. The prevalence of infection varies among *Triatoma* species and across geographic regions [5, 6] and has been reported to be as high as 61% in Louisiana [7].

In the US, *T*. *cruzi* has been found in wild canids; numerous rodent species; and mesomammals such as raccoons, opossums and skunks [1]. The prevalence of *T*. *cruzi* in various wildlife species has ranged upwards from 50% in Texas and some southeastern states [8, 9]. Many of these mammals are peri-urban species that adapt well to human-modified landscapes and, if infected, can bring *T*. *cruzi* closer to humans and their canine companions. In turn, when triatomines are present in the local environment, there may be a subsequent increased risk of vectorial *T*. *cruzi* transmission to people, and an even greater transmission risk to dogs, who likely acquire *T*. *cruzi* via ingestion of infected vectors [10, 11]. In 2006, the Texas Veterinary Medical Diagnostic Laboratory reported 18.6% of 532 dogs presumably clinically ill with cardiac disease to be seropositive for *T*. *cruzi* [12]. In addition, canine serological surveys in states such as Tennessee, Louisiana and Texas indicate that *T*. *cruzi* infection is not an uncommon occurrence, even in apparently healthy domestic dogs [13–15]. Likewise, recent human serological surveys and *Triatoma* blood meal analyses suggest that human *T*. *cruzi* exposure may also occur more frequently than previously realized [16–18]. Physicians and veterinarians are not well-trained to recognize this disease in the US; treatment is not readily available [19]; and there are no drugs approved for veterinary use [1]. Understanding of the ecology of *T*. *cruzi* in the US, including vector and reservoir distribution, and of the molecular epidemiology of endemic strains will enable health and disease control professionals to better respond to the likely rising incidence of Chagas disease.

*Trypanosoma cruzi* taxonomy has been revised, with the most recent consensus classifying the organism into six subtypes or ‘discrete typing units’ (DTUs), designated TcI to TcVI [20]. Within each DTU fall numerous strains whose unique identities are generally determined via typing of several independent genetic loci. Very little *T*. *cruzi* molecular epidemiology research has been done in the US as compared to that accomplished in Latin American countries [21], despite the concern that Chagas may become an emerging disease in the country [19, 22, 23]. Most research on US *T*. *cruzi* has been restricted to typing to the DTU level, and to date, only TcI and TcIV have been detected in local vectors and wildlife [9, 24]. Researchers have recently begun to explore intra-DTU molecular diversity, focusing on isolates from the southeastern US [21]. However, data on genetic diversity in southwestern regions (e.g. California, Arizona, and New Mexico) are very limited [25].

California has the largest influx of migrants of any state in the US [26], with 53% of the immigrant population of Latin American origin [27]. Additionally, 2011 US census data indicates that more than 21% of the nearly 3 million South American migrants residing in the US live in California, with estimates of 75,000–399,000 living in Los Angeles alone [28]. It is therefore probable that many exogenous strains of *T*. *cruzi* enter California every year via human migration. It has been experimentally demonstrated that at least one virulent Honduran strain can be viable if introduced into *Tr*. *protracta*, the most common triatomine bug vector in California [29]. Thus, the pool of *T*. *cruzi* strains present in the US may potentially become more diverse. Additionally, with global climate change, it has been predicted that the human population at risk for *T*. *cruzi* transmission will increase in southern California due to increased triatomine activity associated with warmer temperatures [23]. Therefore, in addition to monitoring *T*. *cruzi* vector distribution, it is important to investigate the molecular genetics of endemic strains; how they compare to virulent strains in Latin America; and whether recently introduced strains may already exist in local vectors. To this end, the goals of this study were to: 1) compare the prevalence and DTUs of *T*. *cruzi* within triatomine bug populations from two regions of California and 2) further characterize the California *T*. *cruzi* samples via molecular genetics to assess whether there are regional differences and to determine how the California samples compare to those present in other regions of the US and Latin America.

## Methods

### Sample collection

*Triatoma protracta* specimens were actively collected from private residences in two study regions. All landowners consented to the collection of bugs from their properties. The first study area was located in southern California, in the town of Escondido (33.1247° N, 117.0808° W). This study site was chosen because previous research had identified *T*. *cruzi* in the resident triatomine bug population [25]. Abundant woodrat (*Neotoma macrotis*) nests were found, and much of the terrain was covered with large granite-based boulders and smaller rocks that provided crevices for triatomines. The second study area encompassed several residences in the town of Vallecito, situated in northern California (38.0903° N, 120.4736° W). This location was selected based on knowledge that multiple triatomine bugs collected there in 2011 were positive for *T*. *cruzi* (M. Niemela, pers comm). Woodrat nests at these properties varied by site but were generally less abundant than the Escondido location.

Black light traps were used in July and August 2012 to collect adult bugs from both study regions. Lights were turned on approximately 30 minutes before sunset and left on for at least two hours after sunset to coincide with the evening hours during which the adult bugs were flying (C. Conlan, pers comm) [30]. The bugs often did not fly the complete distance to the light trap; therefore, combing the surrounding area facilitated capture of bugs crawling on the ground nearby. This trapping method worked well in Escondido, where the trap was strategically placed at the top of a hill and the vegetation on the slope below consisted of small shrubs that did not obscure the emanating light. Light trapping was less successful in Vallecito. Hence, to augment the triatomine sample size from this region, we enlisted the help of property owners to collect bugs found in their homes. We also partially excavated several woodrat nests to obtain both adult and nymphal bugs. All bugs were placed in tubes and frozen at -20C° until laboratory processing.

In addition, we opportunistically obtained specimens from public health employees in southern California, who often received bugs from concerned citizens, especially if the bug had bitten someone within the home. These bugs were shipped to the laboratory either frozen or in ethyl alcohol during the months of April-July 2012 and June-August 2013.

### Laboratory procedures

#### DNA extraction

DNA extractions were performed following the tissue protocol of the QIAamp DNA Blood and Tissue extraction kit (Qiagen). A new razor blade was used to excise the terminal abdominal segments of the *Tr*. *protracta* specimens onto a fresh microscope slide, to which 20 μl of PBS was added before finely chopping the insect tissues. The resulting homogenate was transferred to a 1.5 ml microcentrifuge tube, and the remaining DNA extraction process followed the kit protocol. The final DNA was eluted in 100 μl of AE buffer.

Following the extraction of DNA, polymerase chain reaction (PCR) assays were used to, 1) screen each sample for the presence or absence of *T*. *cruzi* DNA, 2) identify the DTU of each positive sample (i.e. TcI to TcVI), and 3) obtain additional partial gene sequences for phylogenetic analyses. Table 1 lists the primers, reaction and thermocycling conditions (as performed in our lab) and expected amplicon size for all PCR assays. All PCR products were visualized via gel electrophoresis using 1.5% agarose, with the exception of the TcZ nuclear and large subunit ribosomal DNA (LSU rDNA) assays, which were run on 2% and 2.5% gels, respectively. DNA extraction, PCR amplification and cloning procedures were performed in dedicated laboratory spaces to avoid the potential for contamination. Each PCR assay contained a negative water and one or more *T*. *cruzi* positive controls (Y-strain and a sequence-confirmed positive CA sample) as quality control measures.

**Table 1:** *Trypanosoma cruzi* PCR assays used for screening, discrete typing unit (DTU), and phylogenetic analyses.

PCR assay^1^	Forward/Reverse Primers & (Reaction conditions)	bp^2^	Cycling conditions (‘ = minutes;” = seconds)	Cloning^3^
**S:** TcZ nuclear 195bp satellite [31]	TcZ1: 5'-CGA GCT CTT GCC CAC ACG GGT GCT-3' / TcZ2: 5'-CCT CCA AGC AGC GGA TAG TTC AGG-3' (20μl: 1.9mM MgCl_2_, 0.2mM each dNTPs, 0.5μM each primer, 0.25U Taq & 4μl buffer)	188	94° x 5’ (94° x 20”, 57° x 10”, 72° x 30”)^35^; 72° x 7’	1
**S:** kDNA minicircle [32]	121: 5'-AAA TAA TGT ACG GGK GAG ATG CAT GA-3' / 122: 5'-GGT TCG ATT GGG GTT GGT GTA ATA TA-3' (25μl: 1.5mM MgCl_2_, 0.2mM each dNTPs, 0.4μM each primer, 0.625U Taq & 5μl buffer)	330	95° x 10’ (94° x 30”, 58° x 30”, 72° x 1’)^35^; 72° x 10’	1
**DTU:** LSU *rDNA* [33]	D71: 5'-AAG GTG CGT CGA CAG TGT GG-3' / D72: 5'-TTT TCA GAA TGG CCG AAC AGT-3' (25μl: 1.5mM MgCl_2_, 0.1mM each dNTPs, 0.4μM each primer, 1U Taq & 5μl buffer)	varies	94° x 3’ (94° x 1’, 60° x 1’, 72° x 1’)^35^; 72° x 10’	1
**DTU:** *HSP60* [34]	For: 5'-GTG GTA TGG G TGA CAT GTA C-3' / Rev: 5'-CGA GCA GCA G AGC GAA ACA T-3' (50μl: 2mM MgCl_2_, 0.1mM each dNTPs, 0.4μM each primer, 2U Taq & 13.3μl buffer)	432–462 prior to RFLP^4^	94° x 3’ (94° x 30”, 52° x 30”, 72° x 1’)^35^; 72° x 10’	1
**DTU:** Mini-exon [33] ^5^	TC: 5’-CCC CCC TCC CAG GCC ACA CTG-3’ / TC1: 5’- GTG TCC GCC ACC TCC TTC GGG CC-3’ / TC2: 5’-CCT GCA GGC ACA CGT GTG TGT G-3’ (25μl: 1.5 MgCl_2_, 0.2mM each dNTPs, 1μM each primer, 1U Taq & 5μl buffer)	varies	94° x 3’ (94° x 30”, 60° x 30”, 72° x 30”)^27^; 72° x 10’	1
**DTU:** *GPI* [34]	For: 5'-GGC ATG TGA AGC TTT GAG GCC TTT TTC AG-3' / Rev: 5'-TGT AAG GGC CCA GTG AGA GCG TTC GTT GAA TAG C-3' (50μl: 2mM MgCl_2_, 0.1mM each dNTPs, 0.4μM each primer, 2U Taq & 13.3μl buffer)	1264 prior to RFLP^4^	94° x 3’ (94° x 30”, 52° x 30”, 72° x 1’)^35^; 72° x 10’	1
**P:** *COII-ND1* maxicircle [35]	ND3.1A: 5’-GCT ACT ART TCA CTT TCA CAT TC-3’ / COII.2A: 5’-GCA TAA ATC CAT GTA AGA CMC CAC A-3’ (25μl: 1.5 MgCl_2_, 0.2mM each dNTPs, 1μM each primer, 1U Taq & 5μl buffer)	1272	94° x 5’ (94° x 30”, 50° x 30”, 72° x 30”)^30^; 72° x 7’	2
**P:** *TR* nuclear [35]	TR Y2S: 5’-ACT GGA GGC TGC TTG GAA CGC-3’ / TR Y2A: 5’-GGA TGC ACA CCR ATR GTG TTG T-3’ (25μl: 1.5 MgCl_2_, 0.2mM each dNTPs, 0.5μM each primer, 1U Taq & 5μl buffer)	1335	94° x 5’ (94° x 1’, 55° x 1’, 72° x 1’)^30^; 72° x 5’	1
**P:** *RB19* nuclear [36]	RB19F: 5’-GCC TAC ACC GAG GAG TAC CA-3’ / RB19R: 5’-TTC TCC AAT CCC CAG ACT TG-3’ (25μl: 1.5 MgCl_2_, 0.2mM each dNTPs, 0.5μM each primer, 1U Taq & 5μl buffer)	408	94° x 5’ (94° x 1’, 55° x 1’, 72° x 1’)^30^; 72° x 5’	3

^1^Bracketed numbers correspond to the original references for each assay. S = Screening; P = Phylogenetic

^2^The target base pair amplicon sizes; for some assays the size varies by the discrete typing unit as depicted in Fig 1

^3^Cloning methods were M13 PCR amplification followed by ExoSapIT (1), Qiagen MiniPrep method followed by EcoRI digestion (2), or direct sequencing without cloning (3).

^4^See Fig 1 for details on the restriction fragment length polymorphism (RFLP) banding pattern for *HSP60* and *GPI*.

^5^Multiplex PCR assay with three primers.

#### *T*. *cruzi* screening assays

Samples were first screened for the presence of *T*. *cruzi* using the primers TcZ1/TcZ2 [31] and 121/122 [32], which target very high copy number target loci (>10^4^ per parasite). If the results of these assays were discordant, then the assay giving negative results was repeated in triplicate. If at least one of these replicates resulted in a positive band, then the sample was considered positive for that assay. Samples that yielded amplicons of the expected sizes for both sets of screening primers were not confirmed via sequencing. If the target band was only visible for one of the two assays, the DNA from this band was extracted, cloned and sequenced to confirm whether the sample was positive.

#### Discrete typing unit assays

**Fig 1:**
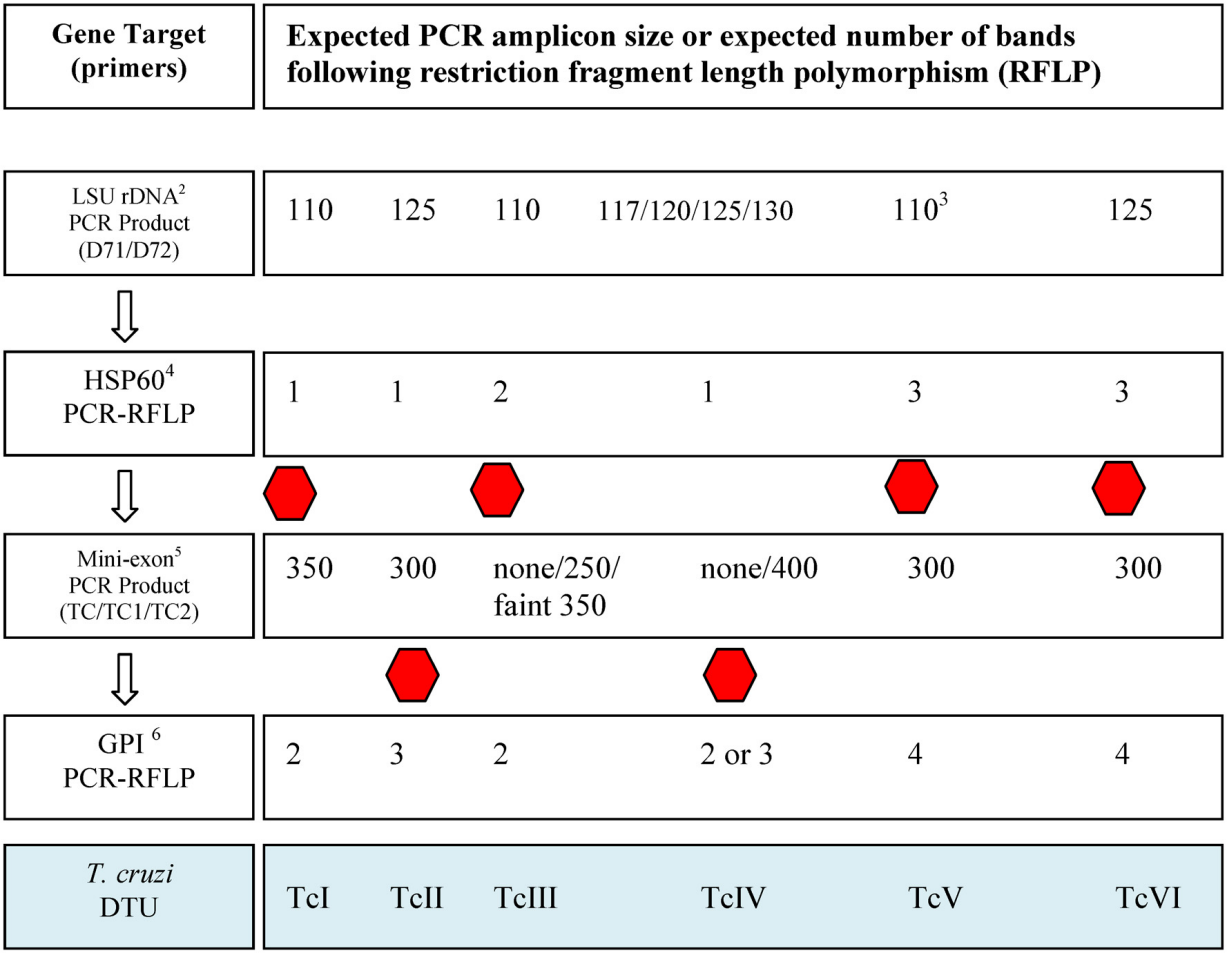
PCR assay flowsheet^1^ to identify *Trypanosoma cruzi* discrete typing units (DTUs). Directional arrows indicate assay order and stop signs denote when sufficient data was gathered to theoretically identify the DTU. The final assay (*GPI*) is included as a confirmatory step, but is not required for DTU identification. ^1^Modified from Lewis et al. [37] ^2^The large subunit rDNA assay is also referred to as the 24sα rRNA gene assay. ^3^An additional band of approximately 125bp may or may not be visible in combination with the 110bp band. ^4^Heat Shock Protein-60 (HSP60) results in an amplicon of 432-462bp, which upon RFLP with *Eco*V restriction enzyme yields the following patterns: 1 band (432–462), 2 bands (118–148 + 314), or 3 bands (118–148 + 314 + 432–462). ^5^This PCR used a pool of three primers to amplify a portion of the non-transcribed intergenic region of the tandemly repeated mini-exon gene. ^6^Glucose Phosphate Isomerase (GPI) results in an amplicon of approximately 1264bp, which upon RFLP with *Hha*I restriction enzyme yields the following patterns: 2 bands (447 + 817), 3 bands (253 + 447 + 490), or 4 bands (253 + 447 + 490 + 817). TcIV will display 2 or 3 bands for North American and South American strains, respectively.

The DTU analysis was performed following a modified version of the triple-assay protocol proposed by Lewis *et al* [37]. As illustrated in Fig 1, the primary PCR assay targets the large subunit ribosomal DNA (LSU *rDNA*) to amplify the D7 divergent domain of the 24S-α rRNA locus using the primers D71/D72 [33]. Based on previous studies, the assay was expected to yield DNA sequences of the following sizes: 110 bp (TcI, TCIII, or TCV); 125 bp (TcII, TcIV, or TcVI); 110 bp + 125 bp (TcV); and 117 bp, 120 bp or 130 bp (TcIV). DNA sequencing analysis was performed on multiple target 110 bp and 125 bp bands to gain representation across both study regions.

The second DTU assay amplified a 432–462 bp segment of the heat-shock protein 60 (*HSP60*). Restriction fragment length polymorphism (RFLP) of this amplicon with EcoRV results in 1–3 bands (Fig 1). TcI, TcIII and TcV, all of which demonstrate a 110 bp band with the LSU *rDNA* assay, are differentiated in the *HSP60* analysis by yielding 1, 2 or 3 RFLP bands, respectively. Likewise, TcII, TcIV and TcVI may all result in a 125 bp band with the primary assay; however, upon RFLP of the *HSP60* sequence, three bands are visualized for TcVI, separating this DTU from TcII and TcIV, both of which only produce a single band.

The non-transcribed intergenic region of the *SL-RNA* (mini-exon) array [33] was used as the third step in the DTU analysis. The mini-exon assay differentiates TcII from TcIV, with 300 bp and 400 bp bands (or no band) amplified, respectively [37]. In this study, the glucose phosphate isomerase (*GPI*) RFLP assay was only occasionally used as a confirmatory test, because it does not differentiate between TcI and the North American TcIV genotype (Fig 1).

#### Phylogenetic marker PCR assays

We selected two single-copy nuclear genes for our phylogenetic analyses, trypanothione reductase (*TR*) and RNA binding-protein-19 (*RB19*). These genes had previously demonstrated strong discriminatory power among both TcI and TcIV isolates [36]. Few published sequences from US *T*. *cruzi* isolates were available for the *TR* and *RB19* genes, and there were no *TR* sequences from TcIV US isolates. However, we expected that the use of *TR* and *RB19* would be more valuable than the inclusion of other nuclear genes previously examined by Roellig et al. [21], which showed low resolution for US isolates. Furthermore, a large number of *TR* and *RB19* reference sequences are available for comparison with isolates from other endemic countries [35, 38–40]. To increase the sensitivity of these PCR assays, bovine serum albumin was added to the PCR buffer at 0.96 mg/ml [41].

We used the primers ND1.3A and COII.2A to amplify a segment of the kinetoplast maxicircle contiguous genes, NADH dehydrogenase subunit 1 (*ND1*) and cytochrome oxidase subunit 2 (*COII*). A major advantage of this gene selection was the availability of homologous sequences for both TcI and TcIV from a broad geographic range, including a large number of isolates from the United States [21]. Secondly, *T*. *cruzi* mitochondrial maxicircle genes undergo a faster mutation rate than do nuclear genes [42]. Therefore these sequences were expected to provide better differentiation both among our isolates and with respect to Latin American strains. Finally, individual parasites contain up to 50 maxicircle copies [42], thus potentially increasing the sensitivity of the PCR as compared to single-copy nuclear genes.

#### Cloning and sequencing methods

The Qiaquick Gel Extraction kit (Qiagen) was used to purify amplicons for either direct sequencing (*RB19*) or cloning (all other assays). Purified products were cloned following the processes described in the TOPO TA cloning kit for sequencing with One Shot TOP10 chemically competent *E*. *coli* cells (Invitrogen).

With the exception of the *COII-ND1* assay, amplification of inserted plasmid DNA was via the colony-PCR method [43]. Selected colonies were transferred directly into individual 200 μl PCR tubes containing the M13 PCR mastermix. Each 1X reaction contained the following: 18.15 μl of PCR grade water, 2.5 μl PCR buffer (10x), 1.5 mM MgCl_2_, 200 μM each dNTP, 0.5 Units of GoTaq DNA polymerase (Promega 5U/μl) and 0.4 μM each of M13 forward (5’–GTA AAA CGA CGG CCA G– 3’) and M13 reverse (5’–CAG GAA ACA GCT ATG AC– 3’) primers. The thermocycling conditions were 94°C (5 minutes); 35 cycles of 94°C (30 seconds), 46°C (30 seconds), 72°C (60 seconds); followed by a final extension at 72°C (5 minutes). PCR products were purified following the USB ExoSAP-IT protocol (USB Products).

*COII-ND1* amplicons were cloned using a modified procedure. First, during the TOPO cloning reaction process, we diluted the vector solution to a total volume of 20 μl, a recommended technique for cloning large inserts [44]. Transformation of cells and plating were performed as above. Several colonies from each plate were selected for additional overnight incubation (37°C) in separate tubes containing 4 ml LB agar broth plus 4 μl of ampicillin (100mg/ml). The tubes were then centrifuged for 5 minutes at 1500 rpm, and plasmid DNA was purified from the pellet using the QIAprep Spin Miniprep kit (Qiagen). EcoRI digestion followed by gel electrophoresis confirmed successful cloning attempts.

All sequencing was performed at the UC Davis Sequencing Lab using ABI Prism analyzer and software. Bidirectional sequences were assembled in Geneious version 5.3.6 (http://www.geneious.com [45]). To confirm that the sequences were derived from *T*. *cruzi*, a comparative search of electronically archived *T*. *cruzi* sequences was run via BLAST in GenBank (http://www.ncbi.nlm.nih.gov/genbank/). The *COII-ND1*, *RB19* and *TR* sequences obtained in this study were submitted to GenBank under the accession numbers KR108801-KR108827 and KR135412-KR135425.

### Phylogenetic Analyses

The program Geneious was used for the assembly and alignment of maxicircle and nuclear sequences. Previously published *COII*-*ND1*, *TR* and *RB19* partial sequences included in our alignments are listed in S1 Table. We prioritized the selection of the *RB19* and *TR* isolates included in the alignment, based on the availability of sequences for both genes, overlap with isolates included in the *COII-ND1* alignment when possible, as well as representation from a broad geographic range.

Two different approaches have been used to amplify the contiguous *COII*-*ND1* maxicircle genes, which span a region of approximately 1,594 bp (CL Brener strain, GenBank #DQ343645). The first approach results in separate partial sequences for each gene, yielding short fragments of 417 bp and 369 bp for *COII* and *ND1*, respectively [42, 46]. The second approach, and the one applied in this study, generates a *COII*-*ND1* combined partial sequence of approximately 1,272 bp in length [21, 35, 39]. The two shorter gene fragments obtained via the first method are completely nested within this longer combined sequence. The goal of our phylogenetic analyses was to maximize the number of unique *T*. *cruzi* sequences included, while ensuring that isolates represented a wide geographic range, especially within the TcI DTU. Therefore the alignment of our *COII*-*ND1* sequences was not limited to published sequences of similar length, but also included the shorter separate gene sequences obtained in studies where the first approach was applied (S1 Table). Following alignment, all *COII-ND1* sequences (n = 62) were trimmed and manually concatenated to a final length of 786 bp (369 + 417), representing the two separate gene fragments.

Phylogenetic trees for the *RB19*, *TR* and *COII*-*ND1* gene sequences were re-constructed in MEGA6 via Neighbor-Joining (NJ) and Maximum Likelihood (ML) methods. In the NJ approach, the evolutionary distances were computed using the maximum composite likelihood method [47] with 2,000 bootstrap replicates. For the ML trees, the best fit model (as determined via the Model Test option in MEGA6) was run with 500 bootstrap replicates. The bootstrap support of the resulting NJ and ML phylogenies were compared for each genetic marker, and the best supported tree was selected. For trees displaying similar topology, both NJ and ML bootstrap values were included at appropriate nodes. In all cases, the trees were outgroup rooted with *T*. *cruzi marinkellei*.

The discovery of an apparent TcI/TcIV hybrid was further evaluated via the comparison of pairwise-distances between this sample and representative samples of TcI and TcIV for each genetic marker (i.e. *T*. *cruzi* sequences included in the reconstruction of the respective phylogenetic trees). The uncorrected p-distances were calculated in MEGA6 using pairwise deletion and transitions/transversions as the substitution types. The program Dna-SP version 5.10 [48] was used to calculate diversity indices for the *TR*, *RB19* and *COII-ND1* TcI sequences obtained in this study. The TcIV sequences were not analyzed due to their limited number. Haplotype diversity (H_d_), nucleotide diversity (P_i_), G+C content and the number of segregating sites (singleton + parsimony informative polymorphic sites) were calculated for all genes. The number of synonymous and non-synonymous mutations, as well as the ratio of number of nonsynonymous substitutions per site to synonymous substitutions per site (dN/dS), were calculated for the *TR* and *RB19* genes but were omitted from the *COII-ND1* analysis due to the putative RNA editing that occurs within the maxicircle gene [49].

## Results

A total of 29 triatomine bugs were collected from the Vallecito study area, of which 24 (two adults and 22 nymphs) were found within woodrat houses. The five remaining bugs were adults obtained from either light traps or within a resident’s home. All identified bugs were *Triatoma protracta*. The two PCR-based screening assays targeting different *T*. *cruzi* genomic loci showed a high degree of concordance in the DNA extracted from all bugs. The 121/122 kinetoplast minicircle assay was marginally more sensitive, detecting 16 positive bugs, whereas the TcZ1/TcZ2 nuclear assay only identified 15 of these same bugs as positive. Kinetoplast DNA sequences obtained from the single discordant sample confirmed the presence of *T*. *cruzi* DNA. Thus 55.2% of bugs at the Vallecito site were infected with *T*. *cruzi*.

At the Escondido study site, 53 bugs were collected, all of which were adult *Tr*. *protracta* drawn to light traps. Thirteen bugs were positive for both *T*. *cruzi* PCR screening assays; however, positive amplification was detected for an additional six bugs using only the kDNA minicircle assay. Five of these discordant samples were successfully cloned and sequenced to confirm the presence of *T*. *cruzi* DNA, yielding the conclusion that 18 bugs (34%) were *T*. *cruzi* positive at the Escondido location.

There were 15 *Tr*. *protracta* bugs submitted from public health employees in southern California, three of which were positive for parasite DNA on both screening assays (20%; there were no discordant results for screening assays among this set of bugs). With the exception of one specimen from San Diego, these bugs represented a range of locations within the Greater Los Angeles Area: Agoura Hills, Altadena, Los Angeles, Northridge, Oak Hills, Santa Clarita, Simi Valley, Tarzana, and Thousand Oaks (S1 Fig). Of the 11 bugs for which addresses were provided, area visualization via GoogleEarth revealed that the homes primarily abutted natural canyon areas designated as parks or were within housing tracts interspersed with parcels of undeveloped land. A summary of the *T*. *cruzi* positive bugs is shown in Table 2. Together these data confirm that wild populations of *Tr*. *protracta* at multiple sites in California are frequently infected with *T*. *cruzi*.

**Table 2:** *Trypanosoma cruzi* results for *Triatoma protracta* specimens. Represented are screening results for all samples tested and discrete typing units (I or IV) for a subset of specimens from each geographic region, based on phylogenetic assays.

Region = % (n/N)^1^	Sample ID^2^	*RB19*^3^	*TR*^3^	*COII-ND1*^3^
Vallecito = 55 (16/29)	Vall3	I	I	I
	Vall7	I	I	I
	Vall8	I	I	I
	Vall13	I	I	I
	Vall14	I	I	I
LA Region = 20 (3/15)	SoCal1	I	I	I
	SoCal2	I	I	I
	SoCal3	I	I	I
Escondido = 34 (18/53)	Esc2	I	I	I
	Esc19	IV	IV	IV
	Esc26^4^	IV	IV	I
	Esc46	I	I	I

^1^Percent bugs positive for each geographic region as determined by highly sensitive nuclear and kinetoplast screening assays (# positive/# tested). LA = Los Angeles.

^2^SoCal1 and SoCal2 were from Los Angeles and Santa Clarita, respectively. The exact location of SoCal3 was unknown. This bug was submitted from a health agency via a wildlife rehabilitation center in Ventura County. An opossum was transferred to the center from an animal shelter in Los Angeles County, and upon arrival, the bug was found in the bedding material within the opossum crate.

^3^Partial gene sequences were submitted to GenBank for RNA binding-protein-19 (*RB19*), trypanothione reductase (*TR*), and cytochrome oxidase/NADH dehydrogenase subunit 1 (*COII-ND1*) under the respective accession numbers: KR108801-KR108812, KR108813-KR108827 and KR135412-KR135425.

^4^Esc26, which displayed incongruency between nuclear (TcIV) and maxicircle (TcI) genes, was categorized as TcI via the DTU assays described in Fig 1.

We next aimed to investigate which *T*. *cruzi* subtype/DTUs were present using lineage-specific genotyping on a subset (n = 29) of the positive bugs, as described in Fig 1. Within this subset, DTU determination was successful only for those samples that were positive for both of the *T*. *cruzi* screening assays described above (n = 22). Samples that were parasite positive only for the more sensitive 121/122 assay (n = 7) likely had insufficient DNA to amplify the lower copy number DTU gene targets. We detected 13 and 7 TcI samples from the Vallecito and Southern California locations, respectively. We found only two TcIV samples, both of which were from the Escondido location. Thus, the *T*. *cruzi* TcI and TcIV DTUs are both endemic in California.

**Fig 2:**
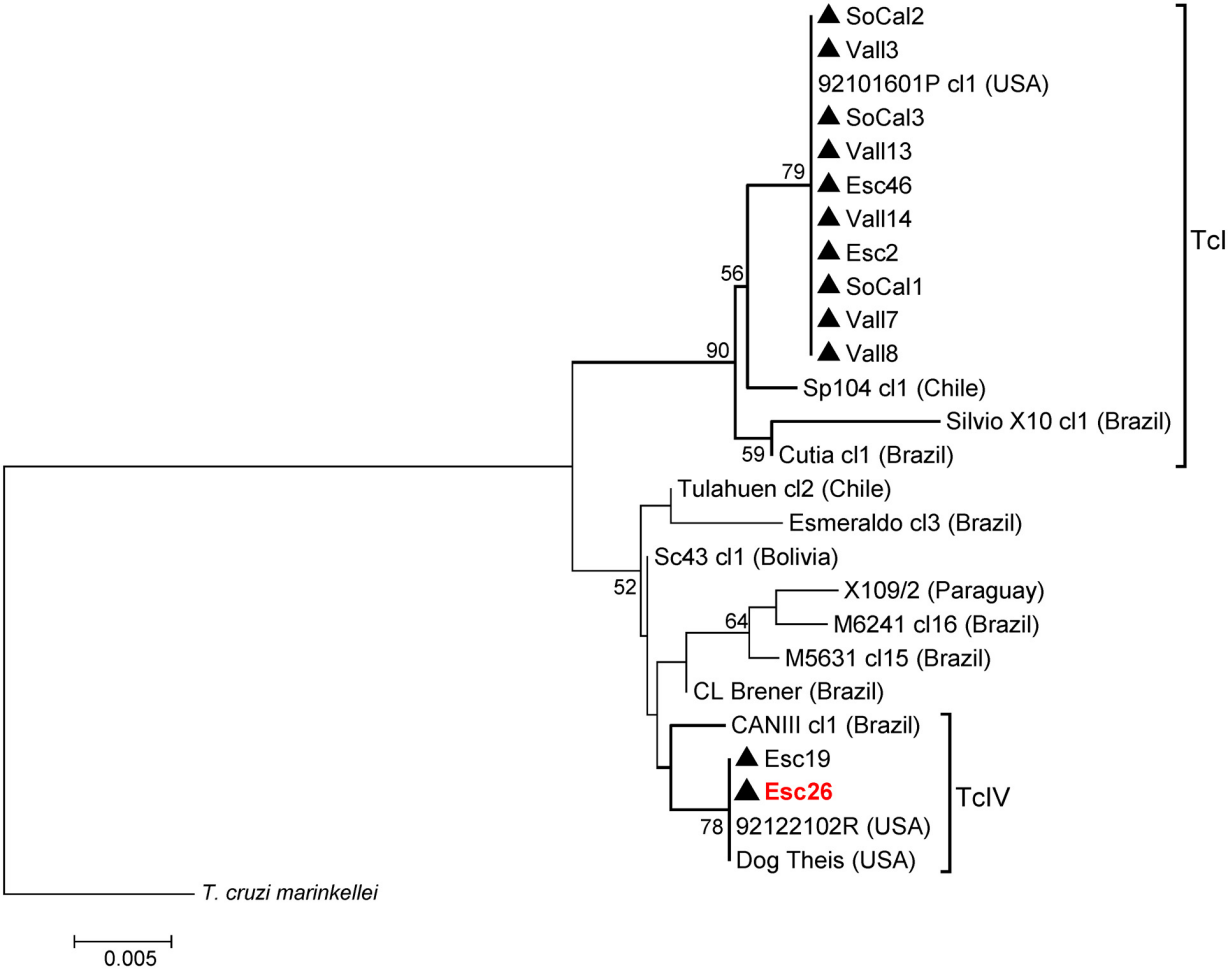
Phylogeny for 26 *Trypanosoma cruzi* 350 bp RNA-Binding Protein-19 (*RB19*) sequences. Neighbor-Joining tree constructed in MEGA6 with evolutionary distances computed via Maximum Composite Likelihood. The numbers above the nodes represent bootstrap confidence levels for 2,000 replicates. Only values ≥ 50% are shown. The only TcI isolate not obtained in this study is represented by a square. Sequences obtained in this study are indicated by a triangle (Esc = Escondido, SoCal = Southern California, Vall = Vallecito). All other isolates represent published GenBank sequences as listed in S1 Table with their country origin indicated in parentheses. The scale bar indicates the number of nucleotide substitutions per site. Tree is outgroup rooted with *T*. *cruzi marinkellei* (TcMark CONTIG 1404).

**Fig 3:**
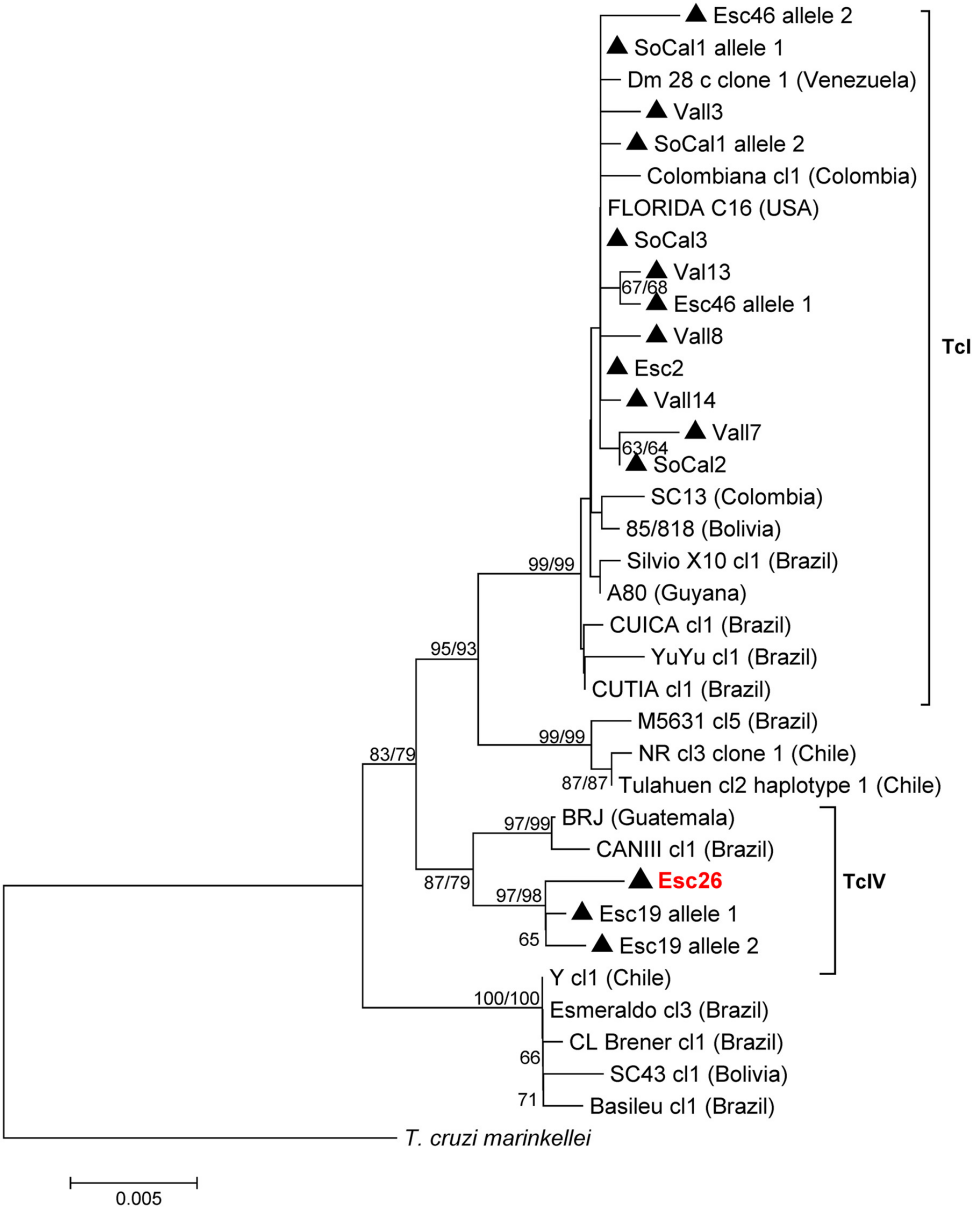
Phylogeny for 35 *Trypanosoma cruzi* 1288 bp trypanothione reductase sequences. Depicted is the Neighbor-Joining (NJ) tree constructed in MEGA6 with evolutionary distances computed via the Maximum Composite Likelihood method and the scale bar indicating the number of nucleotide substitutions per site. The numbers above or below the nodes represent the bootstrap confidence levels for 2,000 NJ replicates (1^st^ value) and 500 Maximum Likelihood replicates (2^nd^ value presented at nodes with congruent topologies) run under the Kimura 2-parameter model for those values **≥** 50%. Sequences obtained in this study are indicated by the triangles (Esc = Escondido, SoCal = Southern California, Vall = Vallecito). All other isolates represent published GenBank sequences as listed in S1 Table with their country of origin indicated in parentheses. Tree is outgroup rooted with *T*. *cruzi marinkellei* (GenBank #AF359007).

The *T*. *cruzi* DTUs that we identified are known to contain substantial genetic diversity [35, 50–53]. We therefore generated nucleotide sequence data to investigate our sample diversity at the intra-DTU level and to enable comparison with strains from other studies. Sequences from two nuclear genes (*TR* and *RB19*) consistently classified our Californian (CA) samples into TcI (n = 10) and TcIV (n = 2) DTUs, confirming our previous genotyping results (Figs 2 and 3). The *TR* gene demonstrated greater sequence diversity across CA samples than did the *RB19* gene: thirteen vs. two unique haplotypes identified respectively. The *RB19* gene sequences for the 10 CA TcI samples were identical and indistinguishable from a single TcI sequence from an opossum isolate [38] obtained from the US state of Georgia (Fig 2). Likewise, the TcIV *RB19* sequences were identical for the two CA samples (Esc19 & Esc26), as well as for two other US samples in GenBank (from a dog of unknown origin and a raccoon from Georgia). In contrast, for the *TR* gene, 2 to 8 single nucleotide polymorphisms (SNPs) were detected among both the TcI and TcIV-positive CA samples (Fig 3). The CA samples were distinguished from two GenBank TcIV sequences from Guatemala and Brazil by 11 to 14 SNPs. Within the TcI group, all the sequences from this study were closely related. The most closely related database sequences were from the US and northern South America (Colombia and Venezuela). Three of the southern CA sequences (Esc2, SoCal1 allele 1 and SoCal3) were identical to each other, as well as to an isolate obtained from a bug collected in the state of Florida (GenBank #AF358970) [35]. For the *TR* gene, the phylogenetic reconstruction between the NJ and ML trees was very similar, and bootstrap values for both trees are presented at congruent nodes (Fig 3). In contrast, the NJ and ML topology for the *RB19* gene varied within major clades, and only the NJ tree is represented (Fig 2).

**Fig 4:**
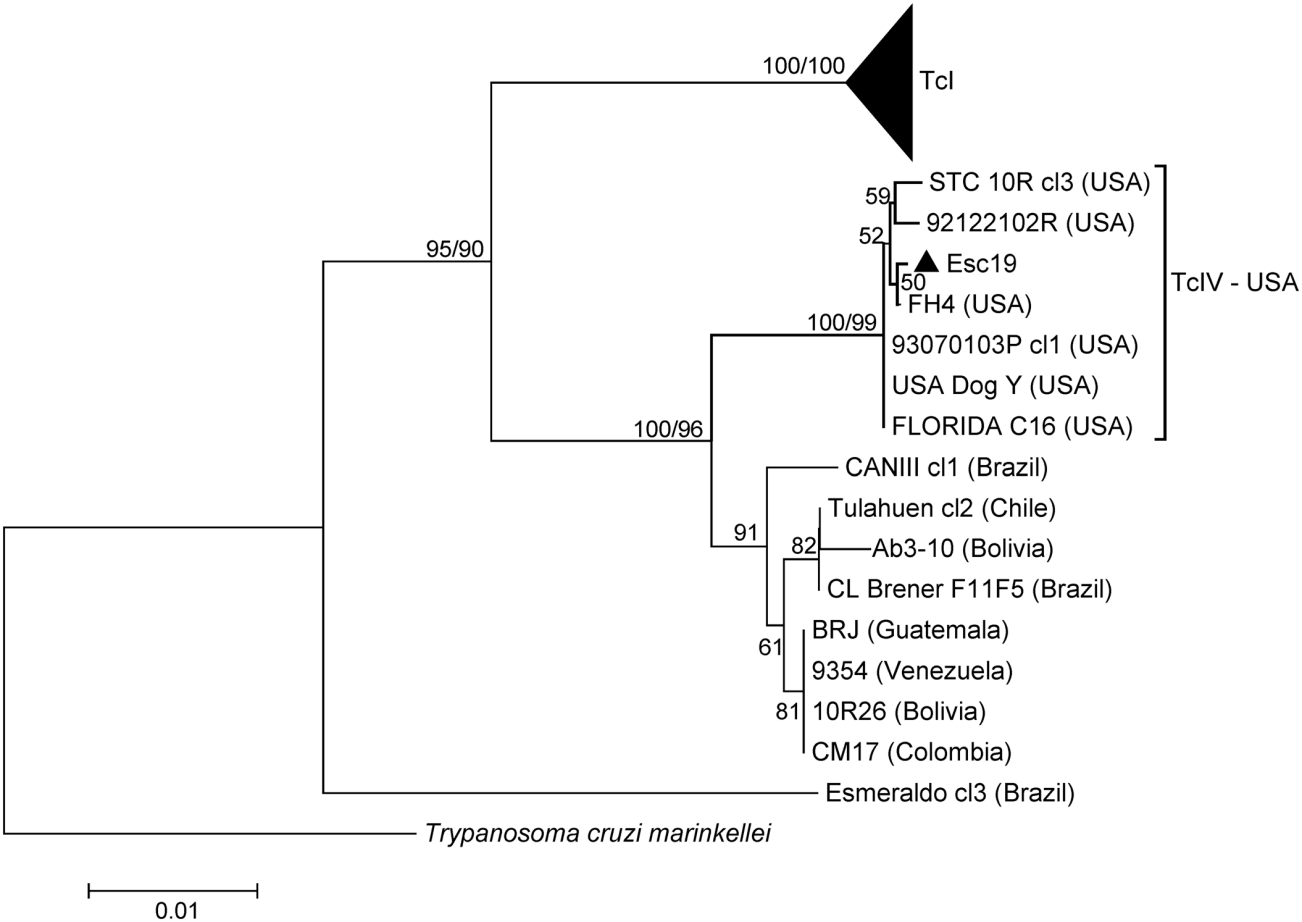
Phylogeny for 62 *Trypanosoma cruzi* concatenated 786 bp cytochrome oxidase II-NADH 1 (*COII-ND1*) sequences. The TcI clade is condensed in this figure and contains the majority of the sequences obtained in this study (see Fig 5 for expanded version). Depicted is the Neighbor-Joining (NJ) tree constructed in MEGA6 with evolutionary distances computed via the Maximum Composite Likelihood method and the scale bar indicating the number of nucleotide substitutions per site. The numbers above or below the nodes represent the bootstrap confidence levels for 2,000 NJ replicates (1^st^ value) and 500 Maximum Likelihood replicates run under the Tamura 3-parameter model (due to slightly incongruent topology, ML bootstrap values are only shown at three nodes) for those values **≥** 50%. Only one sequence obtained in this study (Esc19 = Escondido 19) was grouped as TcIV based on mitochondrial gene sequences. All other isolates represent published GenBank sequences as listed in S1 Table with their country of origin indicated in parentheses. Tree is outgroup rooted with *T*. *cruzi marinkellei* (GenBank #AF359054).

**Fig 5:**
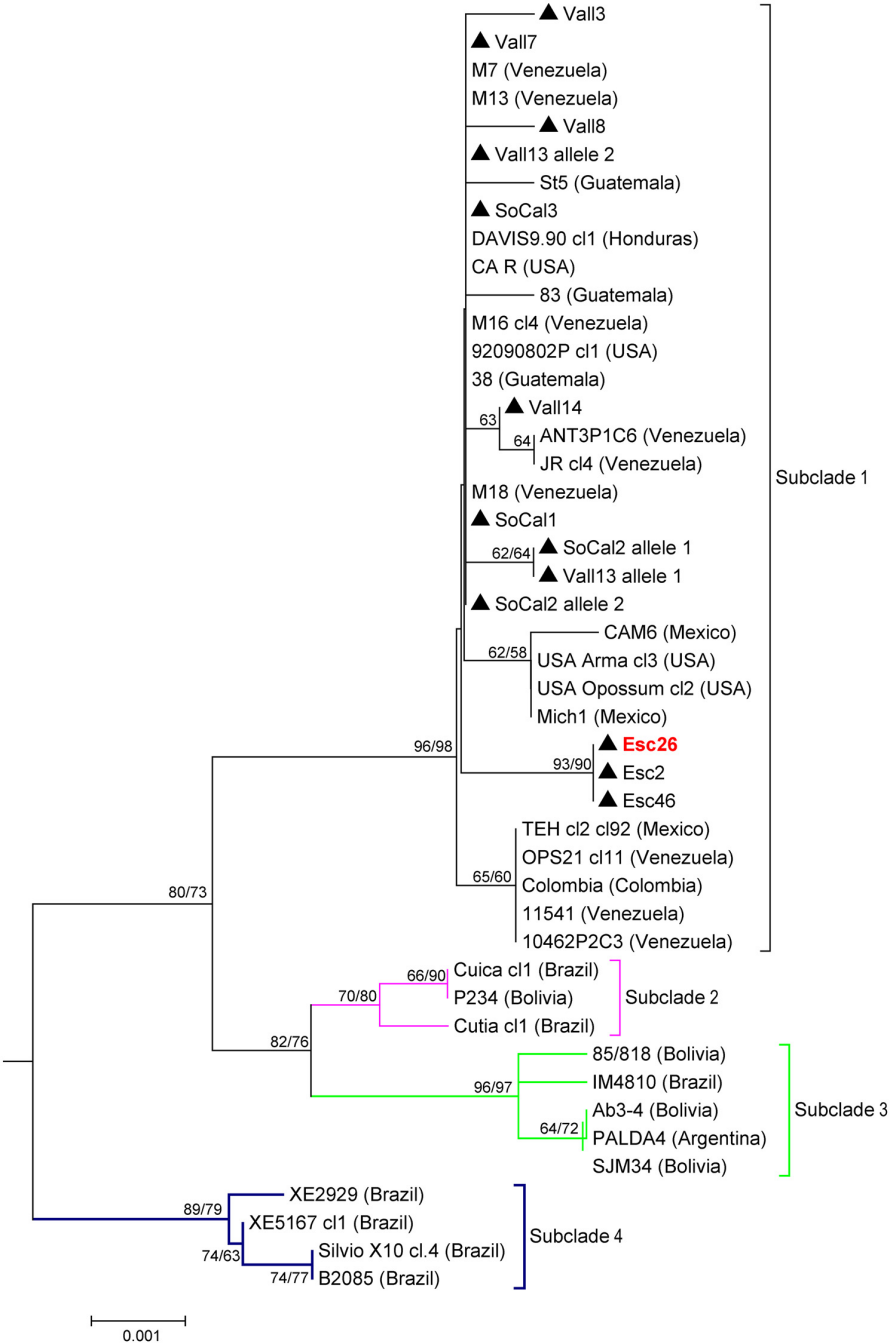
TcI subtree represented as TcI on Fig 4 showing four distinct subclades. This subtree includes 46 *COII-ND1* concatenated sequences (786 bp). The scale bar indicates the number of nucleotide substitutions per site for the NJ tree. The numbers above or below the nodes represent the bootstrap confidence levels for 2,000 NJ replicates (1^st^ value) and 500 Maximum Likelihood replicates run under the Tamura 3-parameter model. Only bootstrap values **≥** 50% are shown. Sequences obtained in this study are indicated by the triangles (Esc = Escondido, SoCal = Southern California, Vall = Vallecito). Esc26 was grouped with TcI in this analysis but was grouped with TcIV in all other analyses. All other isolates represent published GenBank sequences as listed in S1 Table with their country of origin indicated in parentheses.

For the maxicircle *COII-ND1* genes, the NJ and ML tree topologies were very similar within the TcI clade, but the NJ tree provided better support within the TcIV clade. We therefore present the NJ tree with both NJ/ML bootstrap values indicated at congruent nodes (Figs 4 and 5). Eleven of the twelve samples in this study were categorized as TcI based on the analysis of the maxicircle *COII-ND1* genes (Table 2 and Fig 4). Phylogenetic analysis of the concatenated *COII-ND1* sequences revealed that the CA TcI sequences obtained in this study were grouped with strong bootstrap support (96%) in a subclade (Fig 5, subclade 1) with other North American isolates (US and Mexico), as well as several isolates from Central America (i.e. Guatemala and Honduras). In addition, Colombian and Venezuelan isolates previously classified within either “sylvatic” or “domestic” genetic populations [42] were also included in this subclade. Thus, the composition of sequences within subclade 1 closely corresponded to that of the group described elsewhere as TcI-Dom, which contains a high proportion of TcI strains associated with human infection across the Americas [46, 51, 53].

Esc19 was the only sample classified as having a TcIV maxicircle sequence, varying by only 2–3 SNPs from the southeastern US isolates (Fig 4). Interestingly, the Esc26 *COII-ND1* sequence, which was defined as TcIV via the *RB19* and *TR* nuclear gene sequences, as well as the DTU assays, was classified as TcI and was identical to those sequences obtained for Esc2 and Esc46, both of which were typed as TcI by all other markers tested. These data are most consistent with the Esc26 sample being the product of a genetic exchange event between TcI and TcIV ancestors, leading to TcI mitochondrial introgression into a TcIV nuclear genomic background.

The p-distances presented in Table 3 highlight the genetic exchange between TcI and TcIV observed in sample Esc26. With respect to the *RB19* and *TR* markers, Esc 26 was more closely related to TcIV than TcI by an order of magnitude. In contrast, for *COII-ND1*, the reverse finding was apparent. Table 4 provides values for the diversity indices calculated in Dna-SP. As seen in the phylogenetic analyses, no diversity was observed within the TcI sequences for the *RB19* gene, whereas the *TR* and *COII-ND1* genes are more genetically diverse.

**Table 3:** Mean uncorrected pairwise-distances for Esc26. Each p-distance represents the average proportion of nonidentical nucleotide positions between Esc26 and the TcI and TcIV *T*. *cruzi* sequences used to generate the RNA-binding protein-19 (*RB19*), trypanothione reductase (*TR*) and cytochrome oxidase II-NADH 1 (*COII-ND1*) phylogenetic trees. The number of nucleotide positions (bp) for each gene is indicated, as is the number of comparison sequences (n) evaluated within each category.

	TcI		TcIV	
Genetic Marker (bp)	p-distance (SD)	n	p-distance (SD)	n
***RB19* (350)**	0.0198 (0.00174)	14	0.00143 (0.00286)	4
***TR* (1288)**	0.0163 (0.00106)	22	0.00684 (0.00321)	4
***COII-ND1* (792)**	0.00592 (0.00489)	46	0.0769 (0.00192)	6

**Table 4:** Comparative diversity indices for *Trypanosoma cruzi* TcI sequences obtained from *Triatoma protracta* specimens collected in California, USA^1^. The data is represented for the overall combined (Total) populations, as well as at the regional population level (North = Vallecito; South = Escondido and Los Angeles area).

Gene	Pop	bp	No. H	H_d_	P_i_	G+C	S	nSyn mut	Syn mut	dN/dS
*RB19*	Total	368	1 (20)	0.000	0.00000	0.568	0	0	0	n/c
	North	368	1 (10)	0.000	0.00000	0.568	0	0	0	n/c
	South	368	1 (10)	0.000	0.00000	0.568	0	0	0	n/c
*TR*	Total	1288	10 (24)	0.913	0.00231	0.525	17	13	4	1.0150
	North	1288	5 (10)	0.889	0.00304	0.526	11	9	2	1.4032
	South	1288	8 (14)	0.884	0.00770	0.525	8	6	2	0.9379
*COII-ND1*	Total	1225	11 (24)	0.935	0.00287	0.262	18	nd	nd	nd
	North	1225	5 (10)	0.867	0.00214	0.262	10	nd	nd	nd
	South	1225	6 (14)	0.868	0.00227	0.261	8	nd	nd	nd

^1^Table abbreviations: Pop = population region, bp = Base pair alignment length, No. H = number of unique haplotypes (total number of haplotypes analyzed), Hd = haplotype diversity, Pi = nucleotide diversity, G+C = Total G+C content, S = number of segregating sites (singleton + parsimony informative sites), nSyn mut = Number of non-synonymous mutations, Syn mut = Number of synonymous mutations, n/c = not calculable, n/d = not done due to putative RNA editing of this maxicircle gene.

## Discussion

The overall *T*. *cruzi* prevalence of 55.2% (16/29) in the Vallecito triatomine population is the highest reported for *Tr*. *protracta*. This infection level is comparable to that found in *Triatoma gerstaeckeri*, a US triatomine species that was implicated in a 2006 case of acute Chagas disease acquired in Texas [6]. Furthermore, if only the adult *Tr*. *protracta* Vallecito specimens collected in this study are considered, the *T*. *cruzi* prevalence increases to 71.4% (5/7). In contrast, this study’s prevalence of *T*. *cruzi* in adult *Tr*. *protracta* in Southern California (34% for Escondido specifically; 27.9% across all Southern California samples) is consistent with previous infection levels for this species, which have ranged from 20–36% in Southern California [25, 54]. The only published case of locally acquired human Chagas disease in California occurred in Tuolumne County [55, 56], just south of Calaveras County where our Vallecito study site was located. Our data show that *Tr*. *protracta* populations in both northern and southern California have high frequencies of *T*. *cruzi* infection, indicating that the risk for transmission to people and domestic animals is widespread in these regions.

In this research, we successfully amplified *T*. *cruzi* DNA of both mitochondrial (*COII-ND1*) and single-copy nuclear genes (*TR* and *RB19*) directly from *Tr*. *protracta* DNA extracts. Direct testing is commonly done for *T*. *cruzi* screening purposes using highly sensitive assays that target genes possessing thousands of copies (i.e. minicircle kinetoplast targets) or nuclear tandem repeat regions. However, to our knowledge, previous research on the *COII-ND1*, *TR* and *RB19* genes have only used DNA extracted from pure *T*. *cruzi* cultures [21, 36, 40, 42]. Thus it is valuable to note that these assays are sensitive enough for analysis of triatomine bug extracts that have tested positive for *T*. *cruzi* via the TcZ1/TcZ2 assay.

We found greater genetic diversity in Escondido, where both TcI and TcIV DTUs were present as compared to Vallecito where only TcI was detected. Of particular interest is the determination that one of the Escondido samples (Esc26) belonged to TcIV based on DTU and nuclear phylogeny analyses, but that this same sample was grouped with TcI isolates based on the mitochondrial maxicircle *COII-ND1* sequence analysis. To our knowledge, this is the first report of *T*. *cruzi* possessing TcIV nuclear genes and TcI mitochondrial genes. The reverse incongruency, first reported by Machado and Ayala [35] and interpreted as evidence of rare genetic exchange events, has been documented in multiple opossum TcI *T*. *cruzi* isolates in the southeastern US [21].

In the only other molecular typing study performed on *T*. *cruzi* in California [25], samples were obtained from *Tr*. *protracta* specimens collected from two southern sites, including the same suburban property in Escondido from which our samples were collected. In this earlier research, Hwang et al. [25] used the D71/D72 primer set employed in our DTU algorithm to obtain partial sequences of the 24sα ribosomal RNA gene for two samples. Although the authors did not identify these two samples as TcI, alignment of their two sequences (GU594186 & GU594187) with sequences from three of our TcI Escondido samples (KT879367-KT879369) differed by only 2–3 SNPs. It can therefore be inferred that the *T*. *cruzi* samples obtained in the earlier research were also TcI.

With the exception of two samples, our *T*. *cruzi* sequences were all classified as TcI. By contrast, in the southeastern states, Roellig et al. [21] identified twice as many TcIV as TcI isolates. However, this finding may simply reflect host sampling bias and culture success, as isolates were cultured and examined from 21 raccoons versus only nine opossums. In the southeast, raccoons are predominately infected with TcIV, whereas opossums have only been found to be infected with TcI [1, 21]. In California and southwestern states, woodrats are the presumed primary reservoir of *T*. *cruzi*. Research in Texas found that southern plains woodrats (*Neotoma micropus*) were hosts to both TcI and TcIV [9], and it is therefore not surprising that both DTUs were present in our Escondido study site where woodrat nests were extremely abundant. Our study represents the most northerly site of *T*. *cruzi* genotyping to date and therefore extends the known range of TcI. The fact that we did not detect TcIV in our northern site may be due to our smaller sample size in this region or reflect a true absence of this DTU. A geographical distribution for TcIV that extends as far north as Calaveras County remains a possibility; indeed skunks (*Mephitis mephitis*) and raccoons, known hosts of TcIV are present in this region and would be interesting to target for future research.

Our phylogenetic analysis of the concatenated maxicircle genes indicated that the TcI haplotypes from this study clustered within a subclade containing isolates from Venezuela, Colombia, Central America (Guatemala and Honduras) and North America. Included in this subclade were strains from Venezuela (strains 10462P2C3 and 11541) representing a domiciliated *T*. *cruzi* genetic population (VEN_dom_/TcI_dom_) that has been associated with human infections [42, 46]. In a phylogenetic analysis based on nine concatenated maxicircle sequences, the TcI_dom_ strains were similarly nested among North and Central American TcI strains [46]. The authors suggested that these results provided evidence for initial human contact with TcI occurring in North-Central America, with subsequent southerly movement of TcI_dom_ as early colonizing Amerindians migrated south. However this previous analysis included *T*. *cruzi* sequences from only four US isolates, all of which originated in the southeastern US. Thus the placement of all our CA TcI samples in the same *COII-ND1* subclade as TcI_dom_ strains provides further phylogenetic support for the close relationship of US TcI isolates to TcI_dom_ and so strengthens the conclusions proposed by these authors.

The gene sequences examined in this study only represent a fraction of the *T*. *cruzi* maxicircle and nuclear DNA. Genome scale analysis would enable a better understanding of the genetic relationships between strains found in the US and Latin America. Genetic analyses of US *T*. *cruzi* sequences obtained in this study and a limited number of US isolates included in other studies [42, 46] have revealed that US TcI haplotypes are similar, or even identical, to partial gene sequences of several Latin American *T*. *cruzi* strains associated with human illness. Therefore, although some researchers have questioned whether local *T*. *cruzi* strains are as infective or virulent as strains found in Latin America [5, 57, 58], our data suggest there is little genetic basis for considering US strains to have any particular unique characteristics that would distinguish them from Central American or most domestic South American TcI strains. In fact, US *T*. *cruzi* strains have been demonstrated to infect and cause clinical symptoms and pathology in dogs [59–61], non-human primates [62, 63] and humans [56, 64], including a Texas prisoner who contracted acute Chagas disease as a result of an unethical experimental study conducted in 1940 prior to the identification of the first case of autochthonous human Chagas disease in the US [65]. Nevertheless, there are few documented cases of locally acquired human Chagas disease in the US, and it has been proposed that the apparent rarity is most likely due to the infrequency of triatomine colonization within US homes [1, 5, 66] and reduced vector-transmission efficiency [1].

In some regions of Latin America, rural houses are constructed of materials (i.e. adobe brick walls, thatch roofs) that facilitate the invasion and exploitation of breeding niches by triatomines [67, 68]. In contrast, US standard housing construction likely hinders triatomines from establishing breeding colonies once adult bugs have entered homes [69]. However, it is conceivable that outdoor pet enclosures or substandard housing might be subject to colonization events if these structures were adjacent to natural areas with triatomine bug populations, especially if preferred breeding sites and sylvatic hosts had recently diminished. For example, adult *Tr*. *protracta* have been reported to disperse into human residences following the destruction of woodrat nests associated with construction activities [70]. Likewise, following environmental changes in Louisiana spurred by the wake of hurricane Katrina, infestations of *Tr*. *sanguisuga* in human dwellings may have been related to the triatomines’ search for new bloodmeal sources [7].

Emphasis has been placed on *T*. *cruzi* vector-human transmission being less efficient in the US due to the delayed defecation habits of some US triatomine bug species [1]. Yet experimental studies conducted on immobilized mice have demonstrated that two southwestern US species of triatomines, *Tr*. *protracta* and *Triatoma rubida*, may defecate upon repletion, that is, immediately after terminating a blood meal and disengaging from the host [54]. The relevance of these experimental studies with respect to the natural feeding behavior of *Tr*. *protracta* in human homes is not clear; however, given that 75% (6/8) of the observed *Tr*. *protracta* defecations occurred either before or upon repletion, the risk of *T*. *cruzi* vector transmission by this triatomine species cannot be disregarded. Furthermore, although the close ecological associations of some US triatomine species (i.e. *Tr*. *protracta* and woodrats) suggest host feeding preferences, bloodmeal analysis has indicated that vectors are not truly host-specific. In studies performed in California and Arizona, human blood in addition to other host species (e.g. chickens, pigs and wildlife), were identified from three triatomine species: *Tr*. *protracta*, *Triatoma recurva* and *Tr*. *rubida* [17, 71]. Thus, the risk of vectorial *T*. *cruzi* transmission from *Tr*. *protracta* and other US triatomine species is likely to be greater than has been previously assumed.

In fact, recent *T*. *cruzi* screening of blood donors identified 16 asymptomatic cases of *T*. *cruzi* infection most likely acquired from local vector transmission in the US [16]. Data extrapolated from this four-year screening study led the authors to conclude that vectorial transmission is not common within this country. However, one must consider the derivation of these data, which is potentially subject to large sampling bias and thus may not represent the general population. Specifically, *T*. *cruzi* screening was performed on asymptomatic blood donors who presumably felt healthy at the time of blood donation. In contrast, it is probable that people who feel unwell (some of whom may have chronic Chagas disease) do not choose to donate blood. If these people do not seek health care, or if their physicians are unaware of the local risk of Chagas infection, then the disease would go undiagnosed. Currently, Chagas disease is a reportable disease in four states [72], with Texas just having recently listed Chagas as reportable in late 2012 [73]. As these states and others begin to more closely track the incidence of human *T*. *cruzi* infections, the true risk of vectorial transmission should become more apparent.

### Conclusion

Our research is one of only two molecular studies on *T*. *cruzi* in California and the first to investigate this parasite’s genetic diversity in the northern portion of the state. While this study’s prevalence of *T*. *cruzi* in *Tr*. *protracta* populations in southern California (~30%) was similar to earlier findings, an even higher prevalence was detected in our northern California study region. The genetic markers employed in this study allowed us to demonstrate the close similarities between *T*. *cruzi* strains in California and those present in other US states, as well as some Latin American countries. Thus, vectors across California present a clear transmission risk to humans and dogs. Additionally, experimental studies have already proven that *Tr*. *protracta* can sustain a Honduran *T*. *cruzi* isolate [29], and *Triatoma infestans* and *Rhodnius prolixus*, two vectors from Latin America, have been shown capable of harboring US *T*. *cruzi* isolates [4]. Although the divergence and migration of *T*. *cruzi* strains occurred over a period of millions of years [50, 53, 74], in this new era of global connectivity, vectors and the pathogens they carry may unknowingly be transported between countries [75, 76]. Therefore if triatomine vectors efficient in *T*. *cruzi* transmission, and perhaps more readily able to colonize human homes, were to be unwittingly introduced to the US, potential mixing of *T*. *cruzi* strains could occur within and among vector species.

Despite the fact that *T*. *cruzi* has been known to exist in the US for at least 80 years, only four states consider Chagas disease to be reportable. In late 2012, Texas became the fourth state to declare Chagas a reportable disease, a decision preceded by a series of in-state studies and clinical case reports on the disease in both canines and humans. Consistent with Texas, our research implies that some areas of California may have a similar risk for *T*. *cruzi* transmission and suggests that California physicians and veterinary practitioners should consider Chagas disease as a potential cause of cardiac illness in regions where *Tr*. *protracta* populations are evident.

## Supporting Information

S1 Table *Trypanosoma cruzi* sequences used in this study for phylogenetic analyses. List of previously published *T*. *cruzi* sequences (as noted by GenBank accession numbers) used for phylogenetic analyses of the cytochrome oxidase II-NADH dehydrogenase subunit 1 (*COII-ND1*), trypanothione reductase (*TR*), and RNA-binding protein-19 (*RB19*) genes. A negative sign indicates that the isolate was not selected for analysis of that gene sequence. Table contents are ordered first by DTU, next by locality, and finally by strain.
(DOCX)

S1 Fig Location of California *Triatoma protracta* specimen collections. Three counties are highlighted: A = Calaveras, B = Los Angeles, and C = San Diego. Lake Don Pedro is indicated by the star. Northern and southern California sample locations are denoted with blue and red circles, respectively. The inset map of the Los Angeles region marks the exact collection points of nine specimens and illustrates their proximity to vegetated natural areas (i.e. peripheral to the urban centers).
(TIF)
