# Supplementary material for: Molecular Diversity of Trypanosoma cruzi Detected in the Vector Triatoma protracta from California, USA
Source: PLoS Negl Trop Dis. 2016 Jan 21;10(1):e0004291. doi: 10.1371/journal.pntd.0004291 (PMC4721664; doi:10.1371/journal.pntd.0004291)
Supplement: S1 Table — List of previously published T. cruzi sequences (as noted by GenBank accession numbers) used for phylogenetic analyses of the cytochrome oxidase II-NADH dehydrogenase subunit 1 (COII-ND1), trypanothione reductase (TR), and RNA-binding protein-19 (RB19) genes. A negative sign indicates that the isolate was not selected for analysis of that gene sequence. Table contents are ordered first by DTU, next by locality, and finally by strain. (DOCX) [file pntd.0004291.s001.docx]

**Table S1.** ***Trypanosoma* *cruzi* sequences used in this study for phylogenetic analyses.** List of previously published *Trypanosoma* *cruzi* sequences (as noted by GenBank accession numbers) used for phylogenetic analyses of the cytochrome oxidase II-NADH dehydrogenase subunit 1 (*COII-ND1*), trypanothione reductase (*TR*), and RNA-binding protein-19 (*RB19*) genes. A negative sign indicates that the isolate was not selected for analysis of that gene sequence. Table contents are ordered first by DTU, next by locality, and finally by strain.

| **Strain^1^** | **DTU^2^** | **Locality^3^** | **Host/Vector** | ***COII-ND1***^4^ | ***TR*** | ***RB19*** |
| --- | --- | --- | --- | --- | --- | --- |
| PALDA4 | I | Argentina | *Didelphis albiventris* | JQ581312 / JQ581078 | - | - |
| 85/818 | I | Bolivia | *Didelphis* *marsupialis* | AF359026 | AF358982 | - |
| Ab3-4 | I | Bolivia | *Triatoma* *infestans* | EU302216 | - | - |
| P234 | I | Bolivia | *Homo sapiens* | JQ581308 / JQ581074 | - | - |
| SJM34 | I | Bolivia | *Didelphis* *marsupialis* | JQ581317 / JQ581083 | - | - |
| B2085 | I | Brazil | *Didelphis* *marsupialis* | JQ581300 / JQ581066 | - | - |
| CUICA cl1 | I | Brazil | *Opossum* *cuica* *philander* | AF359024 | AF358981 | - |
| **Cutia cl1** | I | Brazil | *Dasyprocta* *aguti* | AF359021 | AF358979 | KF889623 |
| IM4810 | I | Brazil | *Didelphis* *marsupialis* | JQ581303 / JQ581069 | - | - |
| **Silvio X10 cl1** | I | Brazil | *Homo* *sapiens* | EU302222 | AF358975 | KF889622 |
| XE2929 | I | Brazil | *Didelphis* *marsupialis* | JQ581323 / JQ581089 | - | - |
| XE5167 cl1 | I | Brazil | *Didelphis* *marsupialis* | JQ581324 / JQ581090 | - | - |
| YuYu cl1 | I | Brazil | *Triatoma* *infestans* | - | AY785713 | - |
| Sp104 cl1 | I | Chile | *Triatoma* *spinolai* | - | - | KF889624 |
| Colombia(na) | I | Colombia | *Homo* *sapiens* | EU302213 | AY785715 | - |
| SC13 | I | Colombia | *Homo* *sapiens* | - | AF358987 | - |
| 38 | I | Guatemala | *Triatoma dimidiata* | JX431235 / JX431110 | - | - |
| 83 | I | Guatemala | *Triatoma dimidiata* | JX431241 / JX431116 | - | - |
| St5 | I | Guatemala | *Triatoma* *dimidiata* | EU302209 | - | - |
| A80 | I | Guyana | *Didelphis* *marsupialis* | - | AF358976 | - |
| DAVIS9.90 cl1 | I | Honduras | *Triatoma dimidiata* | JQ581302 / JQ581068 | - | - |
| CAM6 | I | Mexico | *Triatoma dimidiata* | JX431248 / JX431123 | - | - |
| MICH1 | I | Mexico | *Triatoma dimidiata* | JX431250 / JX431125 | - | - |
| Teh cl2 cl92 | I | Mexico | *Triatominae* | AF359009 | - | - |
| 92090802P cl1 | I | USA | *Didelphis* *virginiana* | JQ581293 / JQ581059 | - | - |
| 92101601P cl1 | I | USA | *Didelphis* *virginiana* | - | - | KF889627 |
| 9307103P cl1 | I | USA | *Didelphis* *marsupialis* | JQ581294 / JQ581060 | - | - |
| CA R | I | USA (CA) | *Homo* *sapiens* | GU212877 | - | - |
| FLORIDA C16 | I | USA (FL) | *Triatoma* *sanguisuga* | AF359010 | AF358970 | - |
| FH4 | I | USA (GA) | *Didelphis* *virginiana* | GU212882 | - | - |
| USA Armadillo | I | USA (LA) | *Dasypus* *novemcinctus* | GU212880 | - | - |
| USA Opossum | I | USA (LA) | *Didelphis* *virginiana* | GU212879 | - | - |
| 9354 | I | Venezuela | *Homo sapiens* | JQ581295 / JQ581061 | - | - |
| 11541 | I | Venezuela | *Homo sapiens* | JQ581296 / JQ581062 | - | - |
| 10462P3C3 | I | Venezuela | *Homo sapiens* | JX431256 / JX431131 | - | - |
| ANT3P1C6 | I | Venezuela | *Homo sapiens* | JX431259 / JX431134 | - | - |
| Dm 28 c cl1 | I | Venezuela | *Didelphis* *marsupialis* | - | AY785717 | - |
| M7 | I | Venezuela | *Didelphis* *marsupialis* | JQ581304 / JQ581070 | - | - |
| M13 | I | Venezuela | *Didelphis* *marsupialis* | JQ581305 / JQ581071 | - | - |
| M16 cl4 | I | Venezuela | *Didelphis* *marsupialis* | JQ581307 / JQ581073 | - | - |
| M18 | I | Venezuela | *Didelphis* *marsupialis* | JQ581306 / JQ581072 | - | - |

**S1 Table (continued)**

| **Strain^1^** | **DTU^2^** | **Locality^3^** | **Host/Vector** | **COII-ND1**^4^ | **TR** | **RB19** |
| --- | --- | --- | --- | --- | --- | --- |
| OPS21 | I | Venezuela | *Homo sapiens* | AF359020 | - | - |
| JR cl4 | I | Venezuela | *Homo sapiens* | HQ604893 | - | - |
| AB3-10 | II | Bolivia | *Triatoma* *infestans* | EU302218 | - | - |
| **Esm cl3** | II | Brazil | *Homo* *sapiens* | AF359035 | AF358992 | KF889635 |
| Y clone 1 | II | Brazil | *Homo* *sapiens* | - | AY785711 | - |
| M5631 cl5 | III | Brazil | *Dasypus* *novemcinctus* | - | AF358989 | KF889636 |
| M6241 cl6 | III | Brazil | *Homo* *sapiens* | - | - | KF889637 |
| CM 17 | III | Colombia | *Dasypus* spp. | JQ581330 / JQ581096 | AF358990 | - |
| X109/2 | III | Paraguay | *Canis* *familiaris* | - | - | KF889639 |
| 10R26 | IV | Bolivia | *Aotus* spp*.* | JQ581327 / JQ581093 | - | - |
| **CanIII cl1** | IV | Brazil | *Homo* *sapiens* | AF359030 | AF358986 | KF889628 |
| **Tulahuen cl2** | IV | Chile | *Homo* *sapiens* | AF359042 | AF359002 | KF889646 |
| BRJ | IV | Guatemala | *Homo* *sapiens* | EU302217 | EU302257 | - |
| 92122102R | IV | USA | *Procyon* *lotor* | GU212871 | - | KF889629 |
| DogTheis | IV | USA | *Canis* *familiaris* | - | - | KF889630 |
| STC 10R cl3 | IV | USA (GA) | *Procyon* *lotor* | GU212900 | - | - |
| USA Dog Y | IV | USA (CA) | *Canis* *familiaris* | GU212899 | - | - |
| SC43 cl1 | V | Bolivia | *Triatoma* *infestans* | - | AY785721 | KF889643 |
| NR cl3 clone 1 | V | Chile | *Homo* *sapiens* | - | AY785723 | - |
| **CL Brener** | VI | Brazil | *Triatoma* *infestans* | AF359041 | AY785725 | KF889644 |
| Basileu clone 1 | ND | Brazil | *Homo* *sapiens* | - | AY785707 | - |

^1^Strains in bold font were used for analysis of all genes. Clone numbers are indicated as “clX”

^2^Presumed Discrete Typing Unit. ND = not done.

^3^Country of origin with state abbreviations (if known) indicated for USA samples: CA = California, FL = Florida, GA = Georgia and LA = Louisiana

^4^When two accession numbers are provided, they indicate the two sequences that were concatenated with the first being the short *COII* (417bp) the second being the short *ND1* (369bp).
