# Supplementary figures and images for: Molecular Diversity of Trypanosoma cruzi Detected in the Vector Triatoma protracta from California, USA
Source: PLoS Negl Trop Dis. 2016 Jan 21;10(1):e0004291. doi: 10.1371/journal.pntd.0004291 (PMC4721664; doi:10.1371/journal.pntd.0004291)

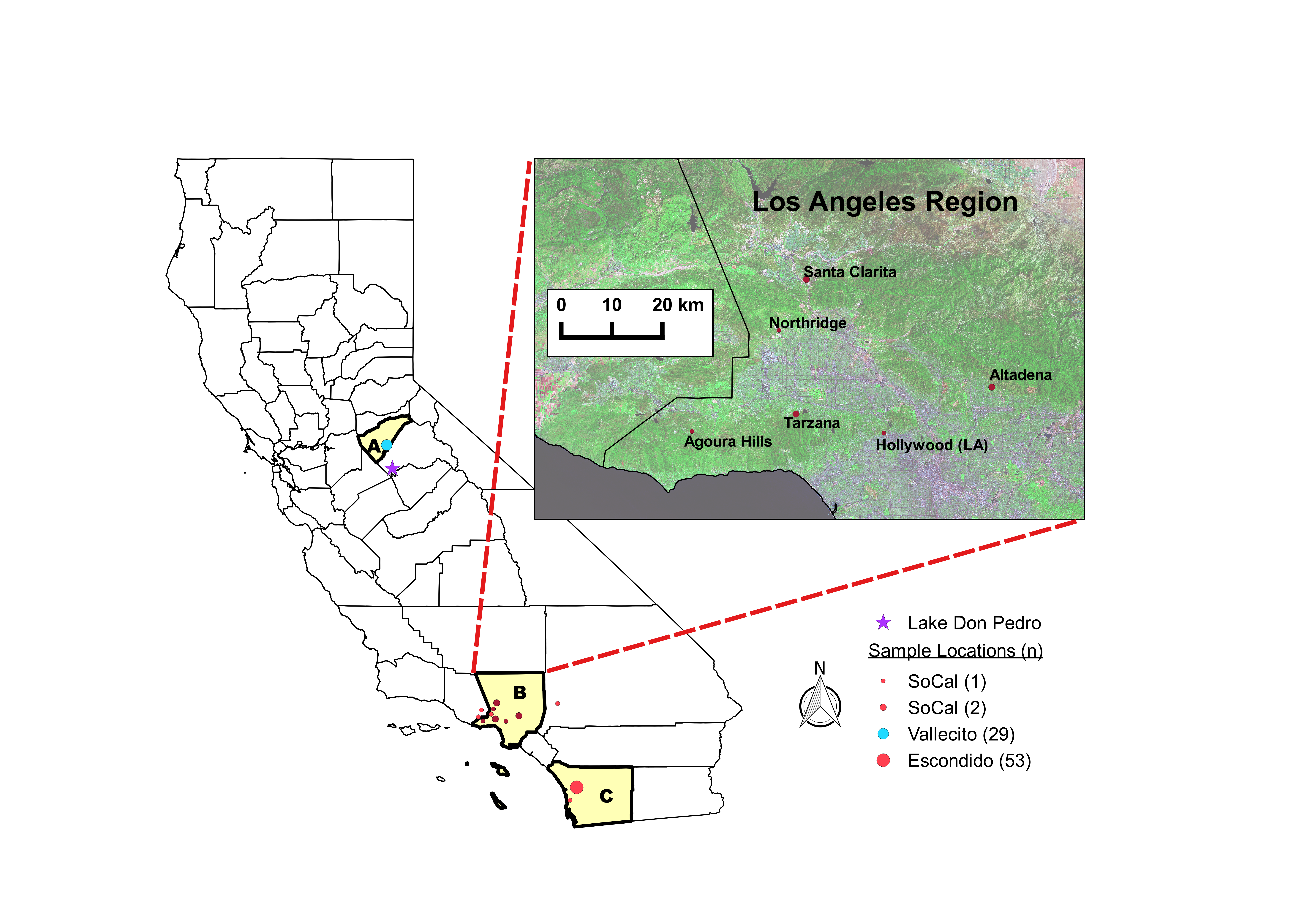

Supplement: S1 Fig — Three counties are highlighted: A = Calaveras, B = Los Angeles, and C = San Diego. Lake Don Pedro is indicated by the star. Northern and southern California sample locations are denoted with blue and red circles, respectively. The inset map of the Los Angeles region marks the exact collection points of nine specimens and illustrates their proximity to vegetated natural areas (i.e. peripheral to the urban centers). (TIF) [file pntd.0004291.s002.tif]
